# Laser-Induced Breakdown Spectroscopy–Visible and Near-Infrared Spectroscopy Fusion Based on Deep Learning Network for Identification of Adulterated Polygonati Rhizoma

**DOI:** 10.3390/foods13142306

**Published:** 2024-07-22

**Authors:** Feng Chen, Mengsheng Zhang, Weihua Huang, Harse Sattar, Lianbo Guo

**Affiliations:** 1 Wuhan National Laboratory for Optoelectronics (WNLO), Huazhong University of Science and Technology (HUST), Wuhan 430074, China; 2 School of Integrated Circuits, Huazhong University of Science and Technology (HUST), Wuhan 430074, China

**Keywords:** LIBS, VNIR, Polygonati Rhizoma, deep learning, adulteration

## Abstract

The geographical origin of foods greatly influences their quality and price, leading to adulteration between high-priced and low-priced regions in the market. The rapid detection of such adulteration is crucial for food safety and fair competition. To detect the adulteration of Polygonati Rhizoma from different regions, we proposed LIBS-VNIR fusion based on the deep learning network (LVDLNet), which combines laser-induced breakdown spectroscopy (LIBS) containing element information with visible and near-infrared spectroscopy (VNIR) containing molecular information. The LVDLNet model achieved accuracy of 98.75%, macro-F measure of 98.50%, macro-precision of 98.78%, and macro-recall of 98.75%. The model, which increased these metrics from about 87% for LIBS and about 93% for VNIR to more than 98%, significantly improved the identification ability. Furthermore, tests on different adulterated source samples confirmed the model’s robustness, with all metrics improving from about 87% for LIBS and 86% for VNIR to above 96%. Compared to conventional machine learning algorithms, LVDLNet also demonstrated its superior performance. The results indicated that the LVDLNet model can effectively integrate element information and molecular information to identify the adulterated Polygonati Rhizoma. This work shows that the scheme is a potent tool for food identification applications.

## 1. Introduction

Polygonati Rhizoma (PR), which is called Huangjing in China, is the rhizome of a liliaceous plant from the genus *Polygonatum* Mill and has been used in traditional food and medicine in China for centuries [1]. PR contains a range of essential compounds such as sugars, lipids, proteins, carotenoids, vitamins, amino acids, and trace elements, which can resist hidden hunger and makes it a potential high-quality crop [2,3]. Rich in compounds like polysaccharides and flavonoids, it offers numerous health benefits, including anti-aging, anti-diabetic, anti-fatigue, and anti-cancer effects [4,5,6,7]. PR has traditionally been used in clinical practices to treat age-related diseases, diabetes, lung diseases, fatigue, feebleness, and indigestion in China, India, Pakistan, Iran, and Japan [4,8]. The wide range of medicinal benefits and the increasing demand for PR in various therapeutic applications underscore the importance of ensuring its authenticity and quality. PR is cultivated in various geographical regions, with China being the main producer. However, the geographical origin of PR affects the quality, drug effect, and price [9,10]. Products certified as protected geographical indications (PGIs) are more popular with consumers and have higher prices. Consequently, unscrupulous traders often mislabel the origins or adulterate PGI products with inferior products or products from other regions to increase profits, causing both healthy and wealthy losses to consumers [9]. In the market, consumers are concerned about whether the product is pure, adulterated, or pure counterfeit. Therefore, the accurate identification of adulterated PR from different geographical origins is essential to protect consumer health and maintain fair trade practices.

Current identification methods for the geographical origin of foods and medicinal materials primarily include manual identification, chromatography, mass spectrometry, and DNA molecular identification [11,12,13,14,15]. However, manual identification requires extensive professional knowledge and is unsuitable for processing products (dry whole root, slice, powder). Chromatography, mass spectrometry, and DNA molecular identification are time-consuming, expensive, environmentally unfriendly, and complicated to operate [15,16,17]. Also, the adulteration of powder samples from different geographical origins has created challenges in these technologies. Therefore, there is an urgent need for a real-time, rapid, direct, efficient, and high-precision method to identify adulterated foods or medicinal materials from different regions.

Currently, some researchers use laser-induced breakdown spectroscopy (LIBS) and near-infrared spectroscopy (NIR) LIBS to identify geographical origin and adulterated foods or medicinal materials products, due to their advantages such as fast and in situ analysis [18,19,20,21]. For instance, Nie et al. employed visible and near-infrared spectroscopy (VNIR) for the quantitative analysis of the adulteration of *Sophora flavescens* powder or corn flour in Notoginseng powder, yielding a predictive R-squared value within the range of 0.86 to 0.94 [22]. Zhao et al. demonstrated the utility of LIBS in analyzing Chinese yam adulterated with cassava and the rhizome of winged yam, with R-squared values reaching 0.9570 [23]. Akin et al. employed LIBS in the analysis of corn and sorghum flour mixtures, achieving a good R-squared result of 0.965 [24]. Some researchers have also fused LIBS and VNIR to achieve better identification results [25]. For example, Zhao et al. used the fusion of LIBS and hyperspectral imaging (400–1000 nm) data to improve ginseng samples’ geographical origin identification accuracy from 96.9% and 94.75% to 98.8% [26]. Collectively, these studies described above verified the potential of LIBS and NIR techniques in the identification of adulterated samples, especially the fusion of LIBS and VNIR, which has a better effect. Combining the elemental and molecular information obtained from these two techniques makes it possible to achieve a more comprehensive and accurate identification of adulterated materials. The subtle chemical and morphological changes between these materials of the same species but with different geographical origins pose a significant challenge for adulteration identification, and single-modal analysis makes it difficult to achieve a high level of identification accuracy. However, there is no report on the identification of adulterated PR from different geographical origins. For food quality identification, the research on the fusion method of LIBS and VNIR at the atomic and molecular levels is rarely studied.

Since foods or herbal medicines are rich in elemental and molecular information, based on the complementary advantages of LIBS in elemental analysis and VNIR in molecular analysis, together, they can provide a comprehensive assessment of the authenticity of PR. Therefore, the purpose of this study is to propose a deep learning model that effectively combines LIBS and VNIR to improve the accuracy of adulteration identification. We proposed an LIBS-VNIR fusion based on a deep learning network (LVDLNet) to detect adulteration in PR sourced from different regions in this study. The model was explained and verified from different aspects. Finally, the study confirmed that the fusion of LIBS and VNIR was feasible and effective in identifying adulterated PR. This work provides a powerful solution for the efficient, accurate, precise, and robust detection of adulteration, which is expected to enhance the integrity and safety of the food supply chain.

## 2. Samples and Experimental System

### 2.1. Sample Preparation

The highest quality PR, produced in Jiuhua Mountain and its surrounding areas in Qingyang County, southern Anhui, is certified as a PGI in China [6,27]. In this experiment, PR from Qingyang County was adulterated with cheaper PR from Dandong City, Liaoning Province. To ensure the authenticity of the samples, our staff personally collected the PR from their respective regions of origin. After the collection, the samples were cleansed with deionized water to eliminate surface dust and debris. Subsequently, they were sliced to a thickness of approximately 2 mm. These slices were then dried to constant weight at a controlled temperature of 60 °C within an electric blast drying oven (101-0B, Shaoxing Shangcheng Instrument Manufacturing Co., Ltd., Shaoxing, China). The dried samples were subsequently crushed and ground to a fine powder, passing through an 80-mesh sieve to ensure uniformity.

In actual market conditions, there are instances where cheaper Polygonati Rhizoma (PR) is used to completely impersonate more expensive PR from famous origins. There are also situations where the cheaper PR is mixed into the more expensive PR for sale, and a small amount of adulteration is insignificant for counterfeiters. To simulate the market adulteration practices, PR from Dandong City was systematically blended with PR from Qingyang County in incremental proportions ranging from 0% to 100% in steps of 20% (ω/ω). The adulterated samples contained 0, 20, 40, 60, 80, and 100% (ω/ω) adulterated levels. Specifically, the adulteration percentages refer to the weight percentage of PR from Dandong City in the mixture. For instance, a 0% level indicates pure PR from Qingyang County, while a 100% level signifies a mixture composed entirely of PR from Dandong City. Intermediate levels at 20%, 40%, 60%, and 80% represent the respective proportions of the Dandong City PR in the blend. The resulting mixtures were then compacted into pellets, each weighing two grams, using an electric tablet press exerting a substantial pressure of 24 tons over one minute. The pressed pellets, characterized by a thickness of approximately 3 mm and a diameter of 20 mm, were employed for our subsequent analyses. Two replicate samples were made for each concentration gradient to eliminate individual differences in samples. A total of 12 pressed pellets were prepared for measurement without further treatment. To evaluate the robustness of our proposed model, we also prepared another batch of samples by blending PR from Baise City in Guangxi Province with authentic PR from Qingyang County, adhering to the stringent criteria outlined in our previous methodology.

### 2.2. Setup and Measurement

**Figure 1:**
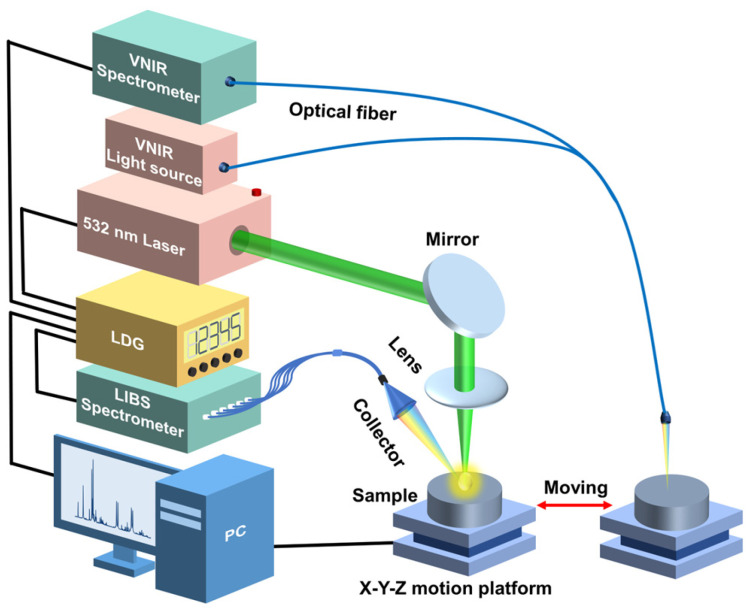
A schematic diagram of the experimental setup.

The schematic diagram of the experimental setup used in this work is shown in Figure 1. This experimental setup mainly consisted of two parts: one was the VNIR acquisition setup, and the other was the LIBS acquisition setup. The VNIR spectra of the samples were collected first. All VNIR spectra were collected using a VNIR spectrometer (QE65pro, spectral ranges: 350–1100 nm; Ocean Optics, Inc., Dunedin, USA) equipped with a Halogen lamp light source (Avalight-HAL-Mini, Avantes B.V., Apeldoom, Netherlands). The samples were placed on an X-Y-Z motion platform (DZY110TA-3Z, Beijing Jiangyun Juli Technology Co., Ltd., Beijing, China) to enable spectral collection at different positions. For the spectral collection process, a precision optical fiber probe was positioned perpendicularly above each sample, thereby enabling the acquisition of the diffuse reflectance spectra. The integration time for each scan was 10 milliseconds, and each spectrum was obtained by averaging ten consecutive scans at each spatial point. In total, 100 distinct spectra per pellet sample were collected at 100 different spatial points. Consequently, for each proportion (adulteration level) of the adulterated samples, we amassed 200 spectra, culminating in a comprehensive dataset comprising 1200 VNIR spectra for six adulteration levels. Each VNIR spectrum had a dimension of 1 × 997.

After the VNIR acquisition, the samples were moved to the LIBS acquisition setup through the displacement platform. For the LIBS acquisition setup, a Q-switched Nd: YAG laser (Beamtech Optronics, Nimma-400; pulse duration: 8 ns; flattened Gaussian beam; Beamtech Optronics Co., Ltd., Beijing, China) operating at 532 nm, 1 Hz, and 130 mJ was used as the ablation source. The laser beam was reflected by a 45° mirror and focused by a quartz lens (focal length: 150 mm) onto the sample surface to generate plasmas. The plasma emission was collected by a collector and transmitted by fiber to a six-channel spectrometer (AvaSpec-ULS4096CL-EVO; spectral ranges: 196–874 nm; minimum gate width: 9 μs; Avantes B.V., Apeldoom, Netherlands). The gate delay and width were set to 2 μs, and 9 μs, respectively. A digital delay generator (LDG 3.0, Wuhan NRD Laser Engineering Co., Ltd., Wuhan, China) synchronized the laser and spectrograph. The experiment was conducted in the air atmosphere. For each pellet sample, 400 spectra were obtained and then averaged to 100 spectra to improve the stability of spectral intensity. Thus, 200 spectra for each proportion and 1200 spectra in total were obtained. Each LIBS spectrum had a dimension of 1 × 24,564.

## 3. Method

### 3.1. The Framework of LVDLNet

**Figure 2:**
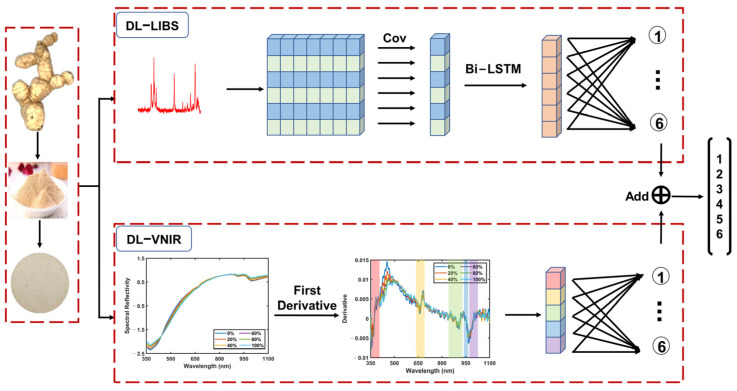
The framework of the proposed LVDLNet. (DL-LIBS: LIBS data processing based on deep learning; DL-VNIR: VNIR data processing based on deep learning; Cov: convolution; Bi-LSTM: bidirectional long short-term memory; Add: Add function; A box of a certain color corresponds to boxes or region of the same color; Numbers 1 to 6 represent the classification results of different adulteration levels).

To realize the identification of adulteration, we propose the LVDLNet framework shown in Figure 2. The proposed LVDLNet includes three main parts: DL-LIBS, which extracts element information; DL-VNIR, which extracts molecular information; and the information fusion part.

### 3.2. Element Information Extraction by DL-LIBS

**Figure 3:**
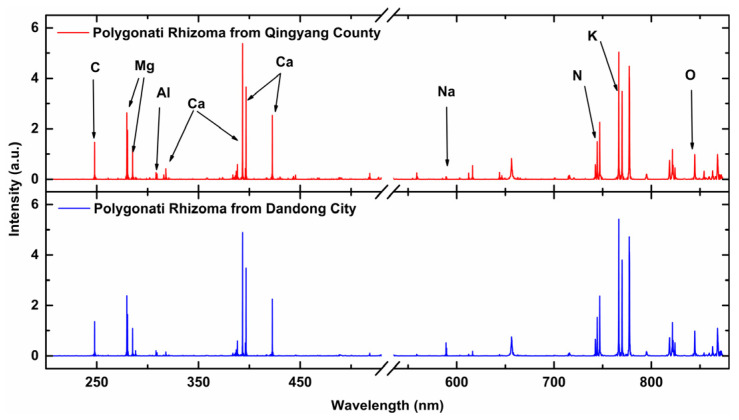
The LIBS spectra of Polygonati Rhizoma from Qingyang County and Dandong City.

The LIBS spectrum can provide element information. The LIBS spectrum lines of PR from two different origins are shown in Figure 3. It can be seen that the LIBS spectral lines of PR from different origins are similar and contain the same elements, with variations primarily in intensity. Distinguishing the origin based solely on individual elemental spectral lines poses challenges, particularly when PR from different sources is mixed. Hence, employing chemometrics becomes essential for discerning these differences.

**Figure 4:**
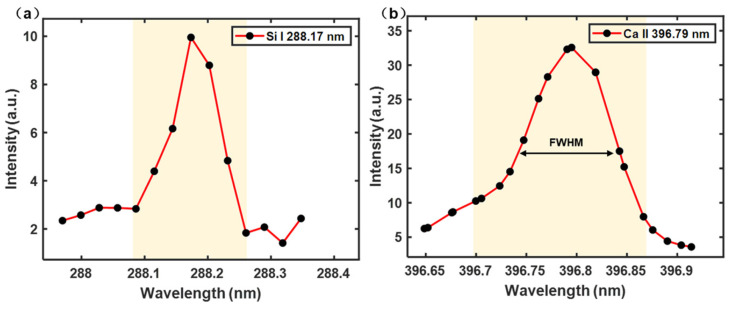
The interval of two typical elemental lines: (**a**) Si I 288.17 nm and (**b**) Ca II 396.79 nm. (The yellow area represents the coverage of the selected element spectral line waveform interval; FWHM: Full Width at Half Maximum).

The characteristic dimension of LIBS full spectrum is 24,564, which contains much invalid information. To reduce background and noise interference, researchers usually select the spectral peak of the element spectral line for analysis [28,29]. Only a few researchers selected the spectral interval (the interval of spectral line profile), which contains the characteristics of spectral wing and the Full Width at Half Maximum (FWHM) [30]. Selecting only the spectral peak will lead the loss of some effective information. Therefore, the selection of a spectral line profile interval is considered in this study. Specifically, for the analysis of PR, we have identified 18 elemental spectral lines with robust signal quality. These lines, with varying numbers of data points across their waveform intervals, are detailed in Table 1. It is observed that each spectral line is characterized by a unique distribution of points, with an average of approximately 14 points. Figure 4 illustrates the waveform intervals for two typical elemental lines: (a) Si I 288.17 nm, which comprises 7 points; and (b) Ca II 396.79 nm, which includes 22 points. Notably, the central 14 points of Ca II 396.79 nm can contain almost the entire waveform of the spectral line, exceeding the FWHM while retaining the critical information for analysis.

**Figure 5:**
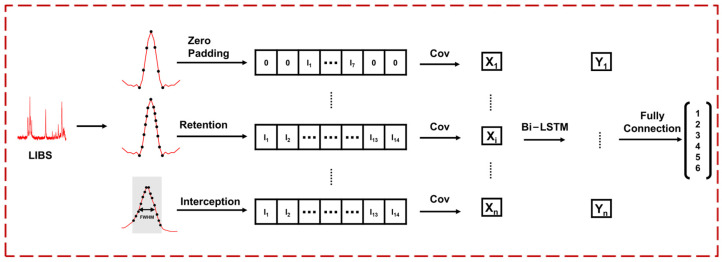
The framework of DL-LIBS. (LIBS: Laser-induced breakdown spectroscopy).

To extract elemental information from the LIBS spectrum effectively, we introduce a deep learning model, as illustrated in Figure 5, termed DL-LIBS. This model initiates the extraction process by performing a convolution operation on the elemental spectral interval to capture the waveform information inherent to each line. To simplify this operation, we standardized the size of the convolution kernel to ensure that it encompassed the complete waveform of each spectral line. Given that 14 data points can contain almost the entire waveform of the vast majority of spectral lines, we set 14 as the convolution kernel size. This selection ensured that the kernel width was sufficient to cover the spectral line’s FWHM, thereby providing a robust basis for the convolution operation.

To standardize the data to a consistent set of 14 points, we employed a triad of strategies tailored to varying spectral profiles: (a) Zero Padding: When the spectral interval comprised fewer than 14 data points, we increased the interval with zero values at both sides, thereby expanding the point total to 14; (b) Retention: For intervals that naturally aligned with the 14-point criterion, we maintained the existing data points without alteration; (c) Interception: When the spectral interval exceeded 14 points, we selectively extracted a central subset of 14 points, while ensuring that this selection spanned beyond the FWHM to preserve the most representative segment of the spectral line. The treatment of each spectral line interval is also shown in Table 1. These standardization strategies allow for a uniform input into the subsequent stages of the model. After the convolution operation, the 18 result values obtained from the 18 spectral lines were fed into the bidirectional long short-term memory (Bi-LSTM) network. The network can identify the nonlinear relationship between spectral lines, improving the accuracy of element information extraction. Next, the extracted LIBS element information was input into the fully connected layer to achieve preliminary classification.

### 3.3. Molecular Information Extraction by DL-VNIR

**Figure 6:**
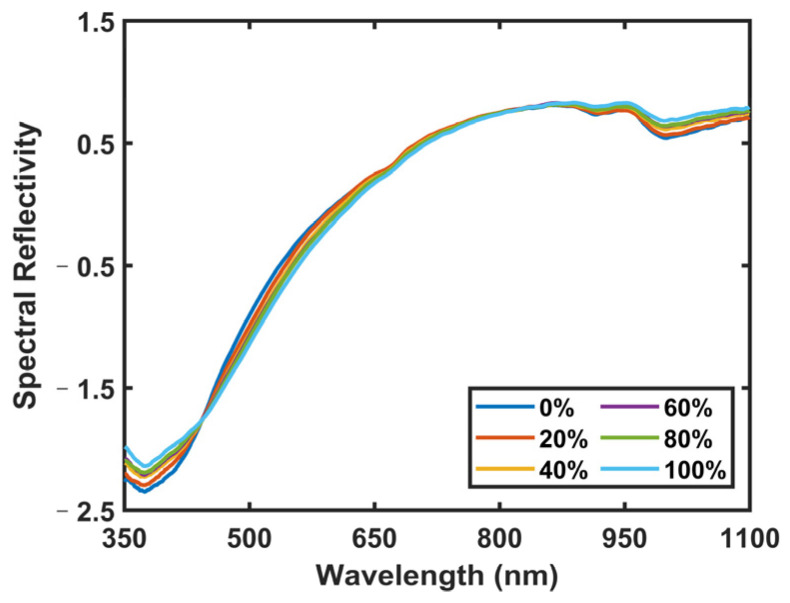
The VNIR spectra of Polygonati Rhizoma with different adulteration concentrations.

Given the challenges in distinguishing mixed adulterated PR using elemental information alone, this study introduces the utilization of VNIR molecular information to augment the identification analysis. The VNIR spectra of PR with different adulteration concentrations are shown in Figure 6. The VNIR spectra of PR elucidate the presence of multiple absorption bands, with the band situated at approximately 670 nm corresponding to the characteristic chlorophyll absorption band [31]. Additionally, the band observed near 920 nm is associated with the second overtone of the O-H stretching vibration, while the band at 970 nm corresponds to the second overtone of another O-H stretching mode [32]. It can be seen that the VNIR spectra with different adulteration degrees have higher similarity and slightly different intensities.

**Figure 7:**
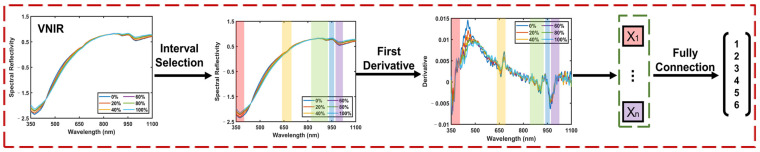
The framework of DL-VNIR. (VNIR: visible and near-infrared spectroscopy; Different colored areas represent different intervals of selection. Boxes of different colors correspond to intervals of the same color).

To extract effective molecular information from the VNIR spectra, we present the framework for the DL-VNIR model in Figure 7. The complexity of near-infrared spectra, characterized by significant overlap and discontinuity, poses a challenge to the direct extraction of component-related information and the subsequent provision of spectral analysis. Unlike discrete points, near-infrared spectra are often manifested as broad bands, a consequence of the myriad vibrational and rotational modes through which molecules interact with light, leading to an extensive array of absorption features. Conventional approaches to feature selection in near-infrared spectroscopy, such as peak and trough detection, have focused on isolated points within these spectral features, often overlooking the information contained within the complete waveform [33,34]. Unlike these traditional methods, our strategy involved an initial selection of the waveband data encompassing both peaks and troughs. The selected intervals of VNIR are shown in Table 2. This selection process targeted five specific wavebands, each representing a significant peak or trough. By employing this refined approach, we effectively condensed the original VNIR data dimensionality from 1044 to 375, retaining valid information while reducing the complexity of the dataset.

Subsequently, we computed the first derivative of the selected intervals, capitalizing on each waveform’s unique degree of change characteristic. We could quantify the absorption variation rate across specific wavelength ranges by utilizing the spectrum slope. This approach effectively mitigated the interference from noise and baseline fluctuations, thereby enhancing the sensitivity of the analysis to subtle changes in sample concentration, composition, or structural attributes [31]. Such optimization is instrumental in elevating the precision of VNIR spectral analysis. The calculated waveform slopes were fed into a fully connected layer. Ultimately, this facilitated the extraction of molecular information and enabled VNIR to preliminary classify the samples.

### 3.4. Information Fusion

In this study, we harnessed the complementary strengths of LIBS and VNIR to perform an analysis of the samples. LIBS provides an in-depth elemental fingerprint, while VNIR offers a detailed molecular profile. Integrating these two modalities is essential for thoroughly understanding the sample characteristics. In the information fusion part, we adopted the Add function to amalgamate LIBS and VNIR data, which is an effective approach in dual-mode data fusion. By combining the data of LIBS and VNIR, this method maintains the integrity and unique attributes of element and molecular information and avoids the possibility of excessive information mixing. The flexibility of the Add function makes it suitable for processing input data of different dimensions and effectively avoids the loss of information [35]. After the operation of the Add function, the Add feature vector was sent to the classification layer to obtain the final results. This final step was crucial as it translated the integrated information into a definitive classification outcome.

### 3.5. Implementation Details

Both LIBS and VNIR obtained 1200 spectra. The dataset was randomly divided into a training set, validation set, and test set according to the ratio of 7:1:2. Thus, the dataset had 840 pairs for training, 120 for validating, and 240 for testing. The training set was used to train the model to establish a prediction model. The validation set was a set of samples left separately during the model training process, which was used to evaluate the performance of the model during the training process and to adjust the parameters and select the model. During the training process, by evaluating the performance of the model on the validation set, the overfitting or underfitting of the model could be found in time, and the hyperparameters of the model could be adjusted according to the results of the validation set. The test set was used to evaluate the final performance and generalization ability of the model. It was a dataset used to simulate the performance of the model in real scenes. All the results presented in this study are test set results. To avoid overfitting, we used data enhancement technology in the training set. Specifically, by adding white noise to the spectral data, and then combining the original spectral data with the spectrum after adding noise, the data expansion of the training set sample was realized. In addition, in the model design, batch normalization and a Dropout layer were added to reduce the risk of overfitting of the model.

In this work, the macro-average evaluation criteria was used to evaluate the model performance. The accuracy (*Acc*), macro-precision (*Mac_P*), macro-recall (*Mac_R*), and macro-F measure (*Mac_F*) were applied as evaluation metrics [36]. *Acc* is the ratio of the number of correctly classified samples to the total number of samples. *Mac_P* is the arithmetic mean of the precision of each category, where the precision is the proportion of the actual positive samples in the predicted positive samples. *Mac_R* is the arithmetic mean of the recall of each category, where the recall is the proportion of the actual positive sample and the predicted positive sample. *Mac_F* is the weighted harmonic average of precision and recall, providing a single score that balances both the precision and recall of the model. These metrics offer a comprehensive assessment of the model’s predictive capabilities and are essential for understanding the reliability of our results. The data processing was carried out on PyTorch 2.0 with a PC of INTEL i7 12700KF CPU (Intel Corporation, Santa Clara, USA), 32G DDR4 RAM (Kingston Technology Corporation, Fountain Valley, USA), and an NVIDIA RTX 3060 GPU (NVIDIA Corporation, Santa Clara, USA)). The size of the GPU was 12 G. The epoch and the batch size were set to 500 and 32, respectively. The learning rate was all set to 0.0005.

## 4. Results and Discussion

### 4.1. Visualization Analysis with t-SNE

**Figure 8:**
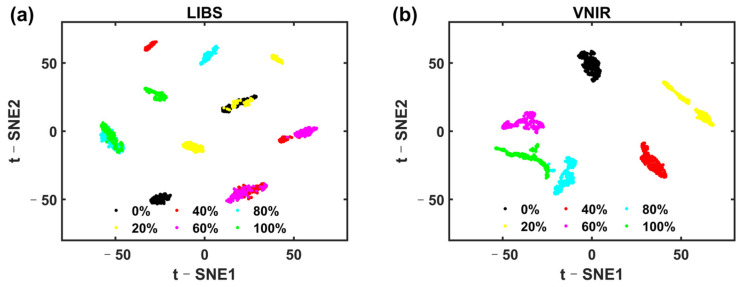
The visualization results with t-SNE based on (**a**) LIBS and (**b**) VNIR.

To observe the clustering effect of different adulterated samples, the full spectral data were visually analyzed with t-SNE. Figure 8 shows the visualization result of t-SNE. It can be seen from the figure that there is a particular clustering effect for different adulterated samples, indicating the feasibility of classification using both LIBS and VNIR data.

### 4.2. Comparison with Different Baseline Models

**Figure 9:**
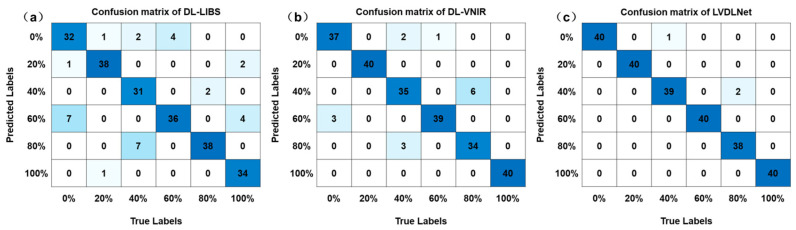
The confusion matrix of baseline modes: (**a**) DL-LIBS, (**b**) DL-VNIR, (**c**) LVDLNet.

In LVDLNet, we used DL-LIBS to extract element information and make a preliminary classification, and DL-VNIR was used to extract molecular information and make a preliminary classification. Then, the two kinds of information were fused to realize the final classification. Table 3 shows the results of a single modality and dual modalities. The four evaluation metrics of DL-LIBS and DL-VNIR were less than 88%, and 94%, respectively. However, the LVDLNet model achieved good results. The *Acc*, *Mac_F*, *Mac_P*, and *Mac_R* of the LVDLNet model were 98.75%, 98.50%, 98.78%, and 98.75%, respectively. The four indicators of the LVDLNet model all exceeded 98%, demonstrating its ability to effectively synthesize LIBS and VNIR data for the enhanced classification accuracy of adulterated PR. Additionally, the confusion matrices for the baseline models are depicted in Figure 9, which facilitates a clear comparison of the classification proficiency between the LVDLNet model and the individual DL-LIBS and DL-VNIR models. For example, in the case of a 0% adulteration level, seven spectral lines from the LIBS data were mistakenly identified as corresponding to a 60% adulteration level, while three spectral lines from the VNIR data suffered the same error. The predictive accuracy for all these lines was successfully rectified upon implementing the fusion process. This further shows that the fusion of LIBS element information and VNIR molecular information can improve classification accuracy.

### 4.3. Effectiveness of Interval Selection

To verify the effectiveness of the selected intervals in this work, the classification effects of different feature inputs were compared. In the comparison, the LIBS spectral peak and intervals were selected as the feature inputs, and the VNIR intervals and full spectra were chosen as the feature inputs. In addition to the DL-LIBS and DL-VNIR models, Principal Component Analysis (PCA) and Support Vector Machine (SVM) were used for verification. PCA and SVM are commonly used in spectral analysis for feature extraction and pattern recognition algorithms, respectively.

The results are shown in Table 4. For DL-LIBS, when the LIBS peak was used as the input, the result was poor, and the four evaluation metrics were about 50%. However, when the LIBS interval was used as the input, the metrics increased to over 87%. For DL-VNIR, the VNIR full-spectra analysis showed slightly higher results than the selected intervals. However, the difference in this result was considered negligible when considering that the intervals with fewer features achieved a classification accuracy similar to full spectra. When using the PCA-SVM model, the spectral interval could also produce better results for LIBS and VNIR data. The above results verify the effectiveness of the selected intervals for the information extraction of LIBS and VNIR in this work. Compared to the LIBS peaks, the LIBS intervals contain more spectral information. Compared to the VNIR full spectra, the selected VNIR intervals essentially contain the information of the full spectrum.

### 4.4. Comparison with Conventional Machine Learning

To evaluate the performance of the model, the result of the deep learning model proposed in this work was compared with the results of conventional machine learning models. The analysis encompassed four distinct conventional machine learning classifiers: Linear Discriminant Analysis (LDA), K-Nearest Neighbors (KNN), SVM, and Extreme Learning Machine (ELM). Similarly, PCA was first used for the feature extraction of selected LIBS and VNIR intervals. After feature extraction, the features were input into the classification model for classification. The classification results for the LIBS and VNIR datasets are presented in Table 5.

Among the four conventional machine learning models, PCA-SVM achieved the best results for both LIBS and VNIR data. However, the deep learning model proposed in this work attained better results. For LIBS data, DL-LIBS improved the results of PCA-SVM from about 82% to more than 87%. For VNIR data, DL-VNIR improved the results of PCA-SVM from about 90% to more than 93%. Moreover, by the fusion of LIBS and VNIR, the LVDLNet model improved the indicators to more than 98%. This demonstrates the superiority of the proposed deep learning model over conventional machine learning technology. Deep learning models can better capture nonlinear relationships in data and adapt to complex and variable data than conventional machine learning.

### 4.5. Universal Verification

To establish the broad applicability of our model, additional verification was conducted using adulterated samples which blended PR from Baise City in Guangxi Province with the authentic PR from Qingyang County. The results are shown in Table 6. As shown in the table, the results of LVDLNet were also better than the single-modality results of DL-LIBS and DL-VNIR. The *Acc*, *Mac_F*, *Mac_P*, and *Mac_R* of the LVDLNet model were 96.25%, 96.25%, 96.30%, and 96.25%, respectively. LVDLNet raised the evaluation indexes from 87% of DL-LIBS and 86% of DL-VNIR to over 96%. This verifies that the models proposed in this study can effectively identify adulterated PR between different geographical origins, proving the effectiveness and robustness of the proposed models.

## 5. Conclusions

Food adulteration identification is essential for protecting consumers’ interests, but no universal method has been widely adopted, especially in industrial scenarios. This study presented a novel deep learning framework, LIBS-VNIR fusion based on a deep learning network (LVDLNet), for identifying adulterated Polygonati Rhizoma (PR). In the LVDLNet model, an interval point standardization strategy in LIBS and a refined peak and trough focus in VNIR data processing improved signal clarity and extraction efficiency. By integrating LIBS elemental information with VNIR molecular information, we enhanced the accuracy of authentication. The LVDLNet model achieved good results, with the accuracy (*Acc*), macro-F measure (*Mac_F*), macro-precision (*Mac_P*), and macro-recall (*Mac_R*) being 98.75%, 98.50%, 98.78%, and 98.75%, respectively. It significantly enhanced the classification evaluation metrics, increasing them from approximately 87% for LIBS and 93% for VNIR to over 98%. Additionally, tests on various adulterated source samples further confirmed the efficacy of the LVDLNet model, with all four classification metrics improved from about 87% for LIBS and 86% for VNIR to above 96%. In addition, this work confirmed the classification effect of the proposed method from different feature inputs and conventional machine learning models. All in all, this study presented a pioneering deep learning framework that synergizes LIBS and VNIR to effectively detect adulterated PR, offering a novel perspective and methodology for identifying food adulteration. Future work can apply this deep learning framework to a wider range of samples, including more different origins and different types of foods, which may be affected by adulteration.

## Figures and Tables

**Table 1:** The LIBS elements’ spectral interval and number of points.

Elements	Wavelength (nm)	Interval (nm)	Points	Operation	Points after Operation
C	247.86	247.72–248.04	11	Zero Padding	14
Mg	280.28	280.16–280.46	11	Zero Padding	14
Mg	285.23	285.08–285.37	11	Zero Padding	14
Si	288.17	288.09–288.26	7	Zero Padding	14
Al	309.29	309.23–309.36	10	Zero Padding	14
Ca	315.89	315.79–315.98	14	Retention	14
Ca	317.93	317.83–318.08	18	Interception	14
Ca	393.34	393.12–393.51	17	Interception	14
Ca	396.79	396.65–396.94	22	Interception	14
Ca	422.64	422.49–422.75	11	Zero Padding	14
Na	588.99	588.90–589.11	14	Retention	14
Na	589.59	589.52–598.71	14	Retention	14
N	742.53	742.30–742.75	9	Zero Padding	14
N	744.35	744.07–744.79	14	Retention	14
N	746.89	746.66–747.38	14	Retention	14
K	766.55	766.23–766.92	14	Retention	14
K	769.89	769.62–770.31	14	Retention	14
O	844.64	844.38–844.96	14	Retention	14

**Table 2:** The selected intervals of VNIR.

Interval (nm)	Points
350.00–400.02	63
639.88–690.76	67
830.92–930.41	136
940.65–960.35	27
966.90–1026.31	82

**Table 3:** Comparison results of baseline models.

Models	*Acc*	*Mac_F*	*Mac_P*	*Mac_R*
DL-LIBS	0.8708	0.8710	0.8780	0.8708
DL-VNIR	0.9375	0.9373	0.9378	0.9375
LVDLNet	0.9875	0.9850	0.9878	0.9875

**Table 4:** Comparison of results under different feature inputs.

Models	Input	*Acc*	*Mac_F*	*Mac_P*	*Mac_R*
DL-LIBS	LIBS Peaks	0.5042	0.4909	0.4783	0.5042
	LIBS Intervals	0.8708	0.8710	0.8780	0.8708
DL-VNIR	VNIR Intervals	0.9375	0.9373	0.9378	0.9375
	VNIR Full Spectra	0.9458	0.9452	0.9450	0.9458
PCA-SVM	LIBS Peaks	0.7875	0.7871	0.7873	0.7875
	LIBS Intervals	0.8250	0.8236	0.8233	0.8250
	VNIR Intervals	0.9000	0.9006	0.9014	0.9000
	VNIR Full Spectra	0.8958	0.8928	0.8969	0.8958

**Table 5:** Comparison results of conventional machine learning and deep learning.

Models	*Acc*		*Mac_F*		*Mac_P*		*Mac_R*	
	LIBS	VNIR	LIBS	VNIR	LIBS	VNIR	LIBS	VNIR
PCA-LDA	0.6083	0.8750	0.6016	0.8732	0.5976	0.8723	0.6083	0.8750
PCA-KNN	0.7625	0.8708	0.7626	0.8708	0.7643	0.8723	0.7625	0.8708
PCA-SVM	0.8250	0.9000	0.8236	0.9006	0.8233	0.9014	0.8250	0.9000
PCA-ELM	0.7333	0.8792	0.7342	0.8757	0.7406	0.8827	0.7333	0.8792
DL-LIBS	0.8708	-	0.8710	-	0.8780	-	0.8708	-
DL-VNIR	-	0.9375	-	0.9373	-	0.9378	-	0.9378
LVDLNet	0.9875		0.9850		0.9878		0.9875	

**Table 6:** Results of universal verification.

Models	*Acc*	*Mac_F*	*Mac_P*	*Mac_R*
DL-LIBS	0.8792	0.8776	0.8777	0.8792
DL-VNIR	0.8625	0.8563	0.8607	0.8625
LVDLNet	0.9625	0.9625	0.9630	0.9625

## Data Availability

The original contributions presented in the study are included in the article, further inquiries can be directed to the corresponding authors.
